# Irisin Attenuates Neuroinflammation and Prevents the Memory and Cognitive Deterioration in Streptozotocin-Induced Diabetic Mice

**DOI:** 10.1155/2019/1567179

**Published:** 2019-06-09

**Authors:** Kexin Wang, Feng Song, Kai Xu, Zhi Liu, Shuhong Han, Fangna Li, Yu Sun

**Affiliations:** ^1^Department of General Surgery, Qilu Hospital of Shandong University, Jinan, Shandong, China; ^2^Department of Orthopedics, Qingdao Municipal Hospital, Qingdao, Shandong, China; ^3^Cheeloo College of Medicine, Shandong University, Jinan, Shandong, China; ^4^Department of Medicine, Division of Rheumatology & Clinical Immunology, University of Florida, Gainesville, FL, USA; ^5^Department of Endocrinology, Qilu Hospital of Shandong University, Jinan, Shandong 250012, China

## Abstract

Diabetes mellitus (DM) patients experience memory and cognitive deficits. The mechanisms underlying this dysfunction in the brain of DM patients are not fully understood, and therefore, no optimized therapeutic strategy has been established so far. The aim of the present study was to assess whether irisin was able to improve memory and cognitive performance in a streptozotocin-induced diabetic mouse model. A diabetic mouse model was established and behavioral tests were performed. We also set up primary cultures for mechanism studies. Western blots and EMSA were used for molecular studies. Significant impairment of cognition and memory was observed in these DM mice, which could be effectively prevented by irisin cotreatment. We also found upregulated levels of GFAP protein, reduced synaptic protein expression, and increased levels of interleukin-1*β* (IL-1*β*) and interleukin-6 (IL-6) in the brains; however, irisin significantly attenuated these cellular responses. Meanwhile, our results demonstrated that irisin inhibited the activation of P38, STAT3, and NF*κ*B proteins of DM mice. Furthermore, our results suggested that irisin might regulate the function of P38, STAT3, and NF*κ*B in hippocampal tissues of DM mice. Collectively, irisin inhibited neuroinflammation in STZ-induced DM mice by inhibiting cytokine release and improving their cognitive function. Our findings revealed the mechanism of irisin's anti-inflammatory effect in the CNS.

## 1. Introduction

Diabetes is a chronic metabolic disorder and is becoming a global public health problem. It results in a significant increase of morbidity and mortality, placing heavy economic burdens on families and health care systems [1]. There is strong evidence that DM increases the risk of cognitive and memory deterioration [2]. Both diabetes and cognitive and memory impairment occur more commonly in elderly people. In DM, one of the prominent pathological changes is hyperglycemia that results from increased glucose production in the liver but decreased insulin production in the pancreas [3]. Such deficits likely contribute to the memory and cognitive dysfunction in DM. Clinical studies have also revealed that many pediatric and young adult patients with DM manifested lower attention and compromised executive functions [4]. Moreover, poor glycemic control in DM accelerated the rate of cognitive decline over time, while improving metabolic control could ameliorate the decline [5]. Meanwhile, around 70%-80% of Alzheimer's disease (AD) patients may have diabetes or abnormal glucose metabolism [6]. The rapid deterioration of cognition in diabetic patients compared to that of nondiabetic patients has been observed in a previous report [7]. These findings especially implied that the consequences of the changes in the central nervous system (CNS) of DM, such as memory and cognition dysfunction, were significantly due to the long-term glucose abnormality. Therefore, it is crucial to decipher the mechanism and develop new strategies to reduce the harm.

Irisin, also known as FNDC5, is a myokine that can increase energy expenditure by accelerating the “browning” of white adipose tissue [8]. Irisin is cleaved from its precursor fibronectin type III domain-containing protein 5 and is associated with better glucose homeostasis by attenuating insulin resistance [8]. Results from some studies have suggested that irisin could be a treatment option for obesity and associated diseases such as type 2 DM [9]. More importantly, recent studies demonstrated that irisin treatment improved endothelial dysfunction in diet-induced diabetic mice [10]. Endothelial dysfunction is a possible underlying mechanism by which memory and cognition deterioration is precipitated in DM animals [11]. Therefore, irisin is a promising candidate to prevent memory and cognition deterioration in diabetic patients.

Astrocytes, the most abundant cell in the CNS, support neighboring neurons and constitute the tripartite synapses [12]. Accumulating evidences have implied that astrocytes play an important role in memory and cognitive function [12]. A recent report demonstrated that repairing damaged astrocytes could improve the cognitive impairments of STZ-diabetic mice [13]. Therefore, it is reasonable to postulate that irisin exerts beneficial effects on DM mice by acting on astrocytes.

The aim of the present study is to assess (a) whether irisin can exert beneficial effects on the neuropathological changes in DM mice, specifically for astrocyte activation, neuroinflammation, and synaptic protein loss; (b) whether the astrocyte is one of the therapeutic targets of irisin; (c) whether irisin is capable of improving memory and cognitive performance and protecting neurons against astrocyte-mediated neuronal damage; and (d) whether irisin can regulate the signaling pathways which trigger the neuroinflammation cascade.

## 2. Materials and Methods

### 2.1. Animals and Drugs

We used 8-week-old male C57BL/6J mice for this animal study. All mice were on a 12 h light : 12 h dark cycle with free access to food and water. Mice were treated according to the guidelines established by the Chinese Council on Animal Care and all procedures were approved by the Animal Care Committee of Qingdao Municipal Hospital, China and Qilu Hospital of Shandong University, China.

Mice were randomly grouped into 4 groups: control, control plus irisin (0.5 mg/kg/day), STZ (150 mg/kg), and STZ plus irisin (0.5 mg/kg/day). Both STZ and irisin were bought from Sigma-Aldrich (MO, USA). STZ was dissolved in distilled 0.1 mmol/l sodium citrate buffer (pH 4.5) and experimental dosages of irisin were prepared in normal saline. We administered a single dose of STZ intraperitoneally to establish a diabetic mouse model. Meanwhile, irisin was given to mice through daily intraperitoneal injection as well. Behavioral tests were performed 3 weeks later.

### 2.2. Y Maze Test

Spatial working memory was assessed by the Y maze test as previously described [14]. The experimental apparatus consisted of 3 arms (35 cm long, 25 cm high, and 10 cm wide, labeled A, B, or C) diverging at a 120° from the central point. The apparatus was placed 40 cm above the floor in a room with dim light. We individually placed mice at the end of the start arm and allowed them to move freely through the maze during an 8-minute session. A mouse was considered to have entered an arm when all 4 paws were positioned in the arm runway. The sequence of arm entries and the total number of entries over a period of 8 min were recorded. The percentage of alternation was defined as the number of sequential triplets containing entries into all three arms (A-B-C and A-C-B constituted a sequential triplet, while A-C-A or A-B-A did not) during the session as a proportion of the maximum possible alternation (equivalent to the total number of arm entries minus − 2) × 100 [14]. Mice whose total entrance number is less than 15 times during the test were not taken into the final data.

### 2.3. Novel Object Recognition

Nonspatial memory was measured using the novel object recognition (NOR) test as previously described [15]. The test was conducted in a Plexiglas activity box (40 cm × 40 cm × 22 cm). Mice were given 15 min daily to explore the empty box for 3 consecutive days prior to the behavioral test. On the fourth day, mice were presented with two identical objects (A1 and A2) for a period of 10 min. After 1 hr, mice were put back into the box and exposed to two objects for 5 min, in which one of the familiar objects (A2) was replaced by a novel object B. Animal activity was considered object exploration if the mice's nose touched the object or if the mice were facing the object within a distance of around 2 cm from the object [15].

### 2.4. Collection of Cerebrospinal Fluid (CSF) Samples

CSF was collected from the cisterna magna, as previously reported with minor modifications [12]. After anesthesia with isoflurane, we made a sagittal incision on the inferior line of the occiput. Then, we separated the subcutaneous tissue and muscles in the surrounding area to clearly expose the meninges. We could collect about 3-4 *μ*l of CSF in the glass tube after penetrating a capillary tube into the translucent meninges.

### 2.5. Western Blot Analysis

Protein samples were boiled and run on SDS-PAGE gels, followed by electrophoretically transferring onto nitrocellulose membranes. The nitrocellulose membranes were then blocked with 5% nonfat dried milk in TBST buffer. The blocked membranes were incubated with an antibody to glial fibrillary acidic protein (GFAP) (1 : 4000; Millipore Corp., MA, USA), synaptophysin (SYP) (1 : 4000, Abcam, Cambridge, United Kingdom), p38 MAPK (1 : 1000; New England Biolabs, Beverly, MA), phospho-p38 MAPK (1 : 1000; New England Biolabs, Beverly, MA), and p-Stat3 (1 : 1000; Cell Signaling Technology, Danvers, MA) and Stat 3 (1 : 1000; Cell Signaling Technology, Danvers, MA) in TBST milk overnight at 4°C. We used *β*-actin as a loading control (1 : 5000; Santa Cruz Biotechnology Inc., CA, USA). Values used for statistical analysis were expressed as the ratio of the band of each protein to the band of their loading control.

### 2.6. Enzyme-Linked Immunosorbent Assay (ELISA)

We measured the concentrations of IL-6 and IL-1*β* in the present study with an ELISA kit (eBioscience, Thermo Fisher Scientific). Each sample was loaded in a duplicate manner with appropriate dilutions to make sure their luminescent units fell within the linear range of standard curves. The values were normalized and expressed as the ratio of each sample to their total loading protein.

### 2.7. Electrophoretic Mobility Shift Assay (EMSA)

EMSA was carried out according to a previous report with a minor modification [16]. Nuclear proteins were extracted from mouse brains. The protein of each sample was probed with excess ^32^P-end-labeled oligonucleotides with a consensus sequence specific for NF*κ*B/DNA (Promega Corp., Madison, WI). The mixture was then fractionated on a 6% polyacrylamide gel for around 2 hours (180 V). The gel was then placed on filter paper and dried. After that, the gel was exposed to a film at 270°C for 1.5 hours followed by autoradiography.

### 2.8. Statistical Analysis

Data in the present study was expressed as the mean ± SEM. The data was then analyzed with one-way ANOVA, followed by the Newman-Keuls *post hoc* test. A *p* value of less than 0.05 was considered statistically significant.

## 3. Results

### 3.1. Irisin Improved the Memory and Cognitive Deficiency in Diabetic Mice

**Figure 1:**
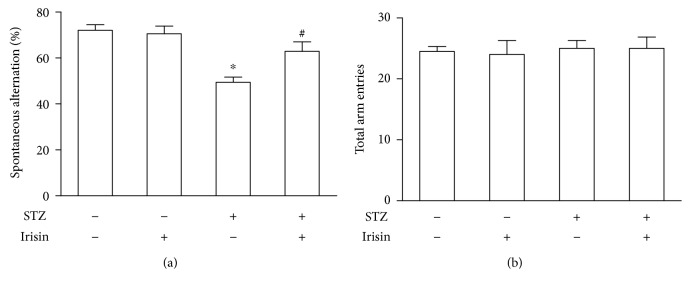
Irisin prevented STZ-induced working memory deterioration in DM mice. (a) STZ treatment caused significantly decreased spontaneous alternation of DM mice in the Y maze test. Irisin could prevent the decrease of the working memory performance of DM mice. (b) Neither STZ nor irisin could change the total arm entries of these mice. All data are expressed as means ± SEM. *n* = 10. ^∗^*p* < 0.05 vs. STZ (-) and irisin (-); ^#^*p* < 0.05 vs. STZ (+) and irisin (-).

**Figure 2:**
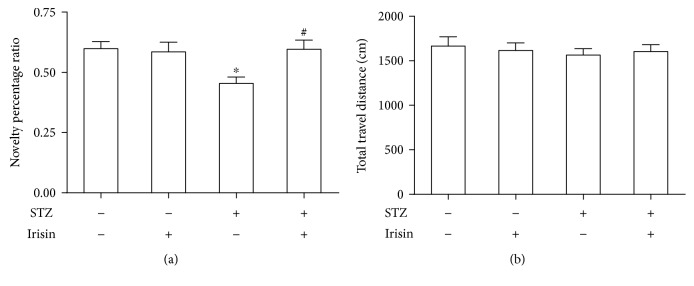
Irisin attenuated memory loss in STZ-induced DM mice. (a) Statistical analysis of the effect of irisin on memory deficits in DM mice. (b) There is no statistical difference of total travel distance during the test among these groups. All data are expressed as means ± SEM. *n* = 10. ^∗^*p* < 0.05 vs. STZ (-) and irisin (-); ^#^*p* < 0.05 vs. STZ (+) and irisin (-).

We tested whether DM mice could demonstrate memory and cognition deficiency in the Y maze and NOR tests. First, short-term spatial memory was investigated with the Y maze. As shown in Figure 1, DM mice showed a significant decrease of alternation behavior compared to the control mice (Figure 1(a)), while the total travel distances were comparable between these two groups (Figure 1(b)). As shown in Figure 1(a), cotreatment with irisin could significantly improve the reduced alternation in DM mice without affecting the total travel distances (Figure 1(b)). These results in the Y maze suggested that short-term spatial memory and spontaneous alternation were significantly reduced in the STZ-induced DM mouse model. Next, nonspatial visual discrimination memory was measured with the NOR test. The NOR test measures the ability of mice to tell between a novel object and a familiar object. To compare the performance of mice in different groups, the time spent interacting with objects (novel or familiar) was measured and a discrimination ratio was used (the ratio of time spent exploring the novel object to the time spent exploring both objects). Mice treated with STZ exhibited a low discrimination ratio (Figure 2(a)). Cotreatment with irisin prevented the STZ-induced memory deficits, while irisin alone did not affect the memory performance of mice (Figure 2(a)). The difference in the behavioral performance was not attributed to alterations in locomotion as the total distance traveled and the total time spent interacting with the objects did not show a significant difference between these groups (Figure 2(b)).

### 3.2. Irisin Inhibited the Increase of GFAP and Prevents Synaptic Protein Loss in Diabetic Mice

**Figure 3:**
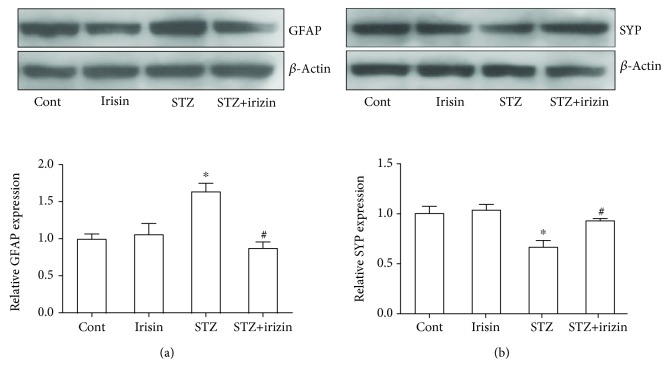
Irisin attenuates the abnormal expression level of GFAP and SYP in DM mouse exposed to STZ. (a) Representative blot picture showing the expression of the GFAP protein in DM mice and the statistical bar graph showing the results. (b) Representative blot picture showing the expression level of SYP in DM mice and the statistical bar graph showing the results. All data are expressed as means ± SEM. *n* = 5. ^∗^*p* < 0.05 vs. Cont; ^#^*p* < 0.05 vs. STZ.

Astrocyte activation is closely related with cognitive dysfunction [12, 17]. We tested with western blot whether the astrocyte activation marker, GFAP, was regulated in these DM mice. As shown in Figure 3(a), STZ treatment induced a significant increase of GFAP protein expression, while irisin could prevent the upregulated protein level. A previous study also suggested that the loss of the presynaptic vesicle protein synaptophysin (SYP) in the hippocampus correlates with cognitive decline in human patients [18]. To understand why irisin improved the behavior of DM mice, brain tissue was processed and measured with western blot to investigate whether irisin could prevent synaptic protein loss in diabetic mice. We found that STZ caused the reduced expression of SYP in DM mice, which could be effectively attenuated by irisin cotreatment (Figure 3(b))

### 3.3. Irisin Attenuated the IL-1*β* and IL-6 Levels in Hippocampal Tissues and CSF of Diabetic Mice

**Figure 4:**
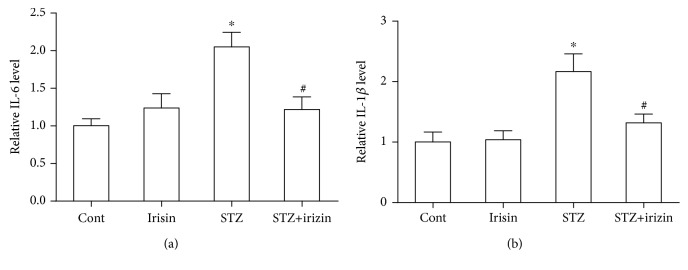
Irisin ameliorated the increased level of IL-6 and IL-1*β* in the hippocampal tissues of DM mice exposed to STZ. (a) Statistical bar graph showing irisin reduced the STZ-induced increased expression level of IL-6 in hippocampal tissues of DM mice. (b) Statistical bar graph showing irisin reduced the STZ-induced increased expression level of IL-1*β* in hippocampal tissues of DM mice. All data are expressed as means ± SEM. *n* = 6. ^∗^*p* < 0.05 vs. Cont; ^#^*p* < 0.05 vs. STZ.

**Figure 5:**
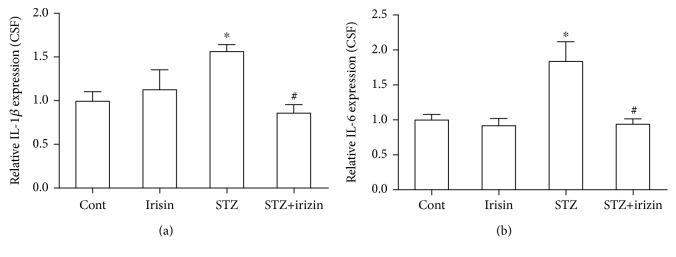
Irisin reduced the level of IL-6 and IL-1*β* in the CSF of DM mice exposed to STZ. (a) STZ increased the level of IL-1*β* in the CSF of DM mice, but irisin treatment could reduce the upregulation of IL-1*β*. (b) STZ increased the level of IL-6 in the CSF of DM mice, and irisin treatment could reduce the upregulation of IL-6. All data are expressed as means ± SEM. *n* = 6. ^∗^*p* < 0.05 vs. Cont; ^#^*p* < 0.05 vs. STZ.

Since we found that irisin could inhibit astrocyte activation in DM mice and improve cognition and memory, we postulated whether neuroinflammation was a response to the neuronal deficiency in these DM mice. Firstly, we tested the expression of IL-1*β* and IL-6 in brain hippocampal tissues of all groups. Our results indicated that STZ caused an obvious increase in IL-1*β* and IL-6 levels in DM, which could be prevented by irisin treatment (Figure 4). Secondly, we tested the level of these two cytokines in the CSF of these mice. We found consistent results of their expression level in CSF (Figure 5). The above results suggested that irisin might attenuate the neuroinflammation in DM mice by reducing the levels of IL-1*β* and IL-6 in their brains.

### 3.4. Irisin Reduced the Activation of P38, STAT3, and NF*κ*B Proteins in the Brain Hippocampal Tissues of Diabetic Mice

**Figure 6:**
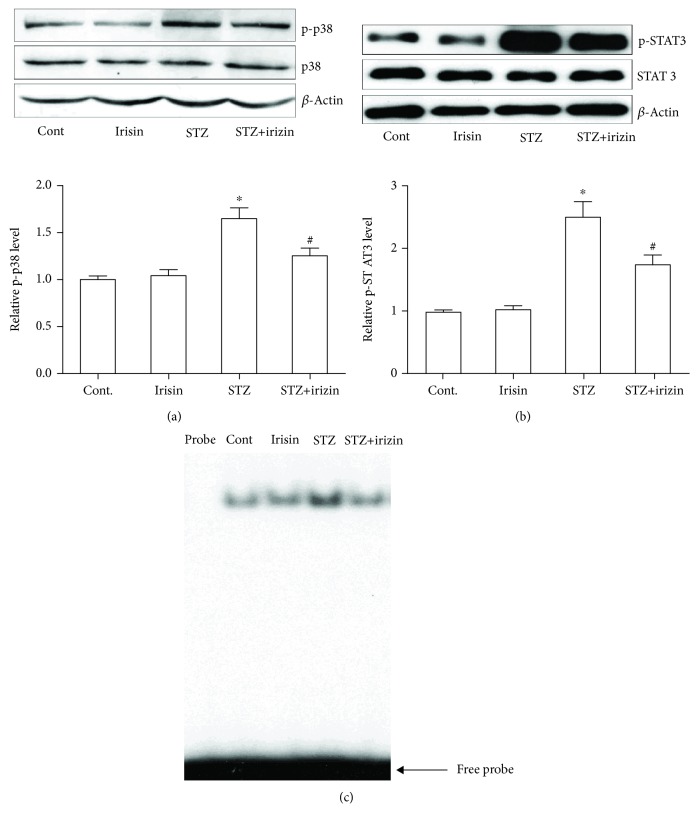
Irisin reduced the activation of p38, STAT3, and NF*κ*B proteins in the brain hippocampal tissues of DM mice. (a) Representative western blot photograph and statistical results of p-p38 and total p38 in hippocampal tissues of all groups. (b) Representative western blot photograph and statistical results of p-STAT3 and total STAT3 in hippocampal tissues of all groups. (c) NF*κ*B/DNA binding activity was determined by EMSA. All data are expressed as means ± SEM. *n* = 5. ^∗^*p* < 0.05 vs. Cont; ^#^*p* < 0.05 vs. STZ.

We also explored the possible underlying mechanism that was responsible for the neuropathological changes in DM mice. Phosphorylation of P38 and STAT3 is closely involved in the cytokine cascade [19, 20]. Therefore, we tested whether the activation of P38 and STAT3 was involved in the response of irisin treatment in these DM mice. The phosphorylation status of P38 and STAT3 was investigated with western blot. We found that STZ caused the increased phosphorylated protein level of P38 in the hippocampal tissues, which could be reduced by cotreatment of irisin (Figure 6(a)). The phosphorylation of STAT3 was measured in the following western blot study. As shown in Figure 6(b), STZ alone induced the upregulated expression level of phosphorylated STAT3 but cotreatment prevented the upregulation. We measured the NF*κ*B activation with EMSA. We found that the binding activity of NF*κ*B to DNA was significantly increased in the brain tissue of DM mice (Figure 6(c)), indicating that NF*κ*B was activated. Treatment with irisin effectively blocked the activation of the NF*κ*B/DNA binding activity in these mice.

## 4. Discussion

Diabetes has detrimental effects on cognitive functioning. These effects are particularly prominent for memory function [21]. However, the underlying mechanisms by which DM affects the CNS remain largely unknown. And therefore, the current therapeutic strategies are far from satisfactory due to the lack of specific medications.

In this study, we explored the possible application of irisin in the treatment of memory and cognitive dysfunction in DM. Diabetic mice were established by treating with STZ, and the effects of irisin on behavioral performances were measured with Y maze and NOR tests. For the first time, our data suggested that irisin could significantly improve the memory and cognitive function in DM mice (Figures 1 and 2). Although irisin is not an approved compound for the treatment of DM, a recent study has provided evidence that irisin could exert therapeutic potential in obesity and type 2 DM by stimulating the “browning” of white adipose tissue [22]. Our findings are consistent with the hypothesis that irisin might be a good candidate for the future treatment of DM since our results here also provided new evidences supporting the therapeutic benefits of irisin by improving memory and cognition in the DM mouse model.

In the CNS, especially in the hippocampus of diabetic mice, an enhanced inflammatory response with astrocyte activation has been observed, which was possibly responsible for the consequent neuronal deterioration [23]. Astrocyte activation was observed in a high-fat feeding animal model [24]. Consistently, we found a significantly increased expression of the astrocyte activation marker protein, GFAP, along with a significantly decreased level of the presynaptic protein, SYP (Figure 3). These results suggested astrocyte activation and synaptic dysfunction in these DM mice. The assumption was further partially supported by our ELISA data with the upregulated expression of two cytokines, IL-1*β* and IL-6, in brain hippocampal tissues and CSF. Interestingly, the above changes in DM mice could be effectively prevented by cotreatment with irisin.

Previous studies have well established the role of p38 and STAT3 in neuroinflammation [25, 26]. In the present study, notable changes of the phosphorylation level of p38 and STAT3 were shown in a western blot study (Figure 6). STAT3 is one of key factors involved in many cytokine cascades, including IL-6, IL-10, and TNF*α* [27]. And a recent study disclosed that the JAK/STAT and STAT3 pathway played a key role in the induction of NMDA-receptor-dependent long-term depression in the hippocampus [28]. Moreover, the STAT3 in astrocytes is an essential step by which astrocytes play a proinflammatory response in the CNS [29]. Our findings here further highlighted the importance of STAT3 in memory and cognitive function, especially in the pathological setting, such as in elderly patients with DM.

To the best of our knowledge, the present study provides the first evidence that irisin prevents memory and cognitive deficits via regulating JAK/STAT and STAT3 and consequent inflammatory injury in the brains of DM mice. We anticipate that our findings here will provide the foundation for future clinical trials to determine whether irisin may prevent or treat memory and cognitive dysfunction in DM patients while attenuating glucose abnormality.

## Data Availability

The data used to support the findings of this study are available from the corresponding author upon request.
